# Defining the Prion Type of Fatal Familial Insomnia

**DOI:** 10.3390/pathogens10101293

**Published:** 2021-10-07

**Authors:** Wiebke Jürgens-Wemheuer, Arne Wrede, Walter Schulz-Schaeffer

**Affiliations:** 1 Institute of Neuropathology, Saarland University Medical Center, Saarland University, 66421 Homburg, Germany; Wiebke.Wemheuer@uks.eu (W.J.-W.); Arne.Wrede@uks.eu (A.W.); 2 Prion and Dementia Research Unit, Department of Neuropathology, University Medical Center Göttingen, Georg August University of Göttingen, 37075 Göttingen, Germany

**Keywords:** fatal familial insomnia, prion disease, prion types, stability, protein aggregates, deposition pattern

## Abstract

Fatal familial insomnia (FFI) belongs to the genetic human transmissible spongiform encephalopathies (TSE), such as genetic Creutzfeldt-Jakob disease (CJD) or Gerstmann-Straeussler-Scheinker syndrome (GSS). Here, we analyzed the properties of the pathological prion protein in six FFI cases by Western blot analysis, a protein aggregate stability assay, and aggregate deposition characteristics visualized with the paraffin-embedded tissue blot. While in all cases the unglycosylated fragment in Western blot analysis shared the same size with sporadic CJD prion type 2, the reticular/synaptic deposition pattern of the prion aggregates resembled the ones found in sporadic CJD type 1 (CJD types according to the Parchi classification from 1999). Regarding the conformational stability against denaturation with GdnHCl, FFI prion aggregates resembled CJD type 1 more than type 2. Our results suggest that the size of the proteinase-K-resistant fragments is not a valid criterion on its own. Additional criteria supplying information about conformational differences or similarities need to be taken into account. FFI may resemble a prion type with its own conformation sharing properties partly with type 1 and type 2 prions.

## 1. Introduction

Fatal familial insomnia (FFI) is a human transmissible spongiform encephalopathy (TSE), such as Creutzfeldt-Jakob disease (CJD) or Gerstmann-Straeussler-Scheinker syndrome (GSS). The always-fatal TSEs or prion diseases are characterized by their experimental transmissibility, spongiform changes in the central nervous system (CNS), and the infectious agent being a misfolded protein (a proteinaceous infectious particle, abbreviated *prion* [1]) that is able to corrupt its physiological counterpart. The less soluble pathological isoform tends to form protease-resistant aggregates. Sporadic, genetic, and acquired forms of prion diseases are known for humans and animals alike. 

Lugaresi et al. described FFI as a genetic disease in an Italian family for the first time in 1986 [2]. Clinically, the disease course is characterized by progressive untreatable insomnia, dysautonomia, endocrine disbalance, and motor signs, while the pathology is dominated by thalamic degeneration [3]. The distribution of pathologic prion protein aggregates is more widespread than the histological lesions characteristic of prion disease, i.e., spongiform changes of the neuropil, neuronal loss, and gliosis [4]. In patients with a short disease course who died during an earlier stage of the disease, the presence of prion aggregates may be limited to the thalamic and midbrain regions, the entorhinal cortex, and the amygdala [4]. A mutation in the prion protein gene resulting in an exchange of aspartate for asparagine at codon 187 (D187N) is responsible for a genetic disease here, but whether the disease phenotype will be FFI (methionine) or genetic CJD (valine) depends on the polymorphism at codon 129 of the affected allele [5]. 

Transmission to mice seemed to be hampered by the comparatively small amounts of pathological prion protein in the (cortical) samples used, but was successful with a thalamic sample from an FFI patient inoculated in NZW mice in the 1990s [6]. Due to the same reasons, detection of pathological prion protein in FFI patients (PrP^FFI^) is often not easy, especially in the living patient when PrP^FFI^ aggregates are sparse in the frontal cortex biopsy site. Conventional immunohistochemistry is often not sensitive enough to detect PrP^FFI^ in formalin-fixed tissue. More sensitive methods such as the paraffin-embedded-tissue blot (PET blot) [7] are able to reveal the same fine reticular prion aggregate deposition pattern as observed in sporadic CJD type 1 [8]. In Western blot analysis, the proteinase K cleaved PrP^FFI^ shows the typical three-banded prion protein pattern with an unglycosylated fragment of 19 kDa [4,9], such as the one in sporadic CJD type 2 [10]. However, the di- and monoglycosylated fractions are more pronounced in FFI than in sporadic CJD, thereby giving FFI its own characteristic Western blot profile [11]. 

The finding that obvious parallels exist between CJD types and ovine scrapie types regarding deposition pattern and prion stability suggests that prion types are present across species [12]. Here, the complex prion deposits of sporadic CJD type 2 and classical scrapie both result in a higher stability toward GdnHCl than the reticular, synaptic prion aggregates of sporadic CJD type 1 and the atypical/Nor98 scrapie. The term “prion type” refers to properties of the disease agent itself, and the term “prion strain” includes host-associated factors that interact with the disease agent upon transmission and may moderately modify the clinical disease presentation. Strain factors can be discovered only after transmission and passaging of the agent in a host of a different species. The correct use of terminology allows a differentiation between disease agent and host factors [13]. In the current study, we aimed to characterize the pathological prion aggregates in six FFI patients more closely. For this purpose, we used sensitive methods such as the PET blot technique. For frozen tissue, we applied a membrane adsorption assay (MAA) to detect prion aggregates and their stability against GdnHCl, and Western blot analysis [14]. FFI might belong to the type 1-type 2 classification schemes or could resemble a type on its own. 

## 2. Results

**Figure 1:**
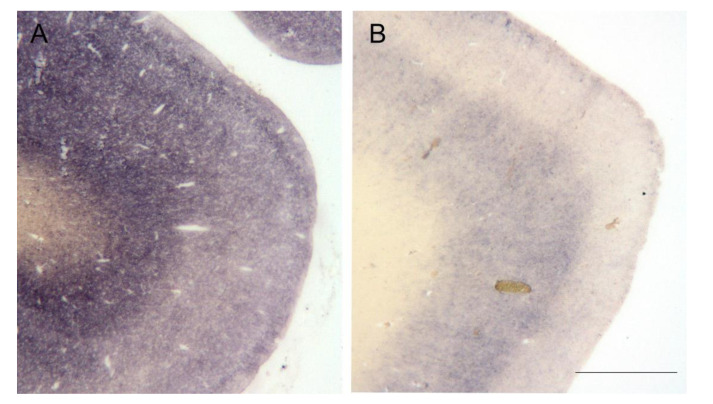
Pathological prion aggregates (PrP^FFI^) detected in the cingulate gyrus of patient #4 (**A**) and patient #2 (**B**) with the PET blot using mAb 12F10 1:5000 (bar = 1 mm). While patient #4 (**A**) displays large amounts of synaptic PrP^FFI^, prion aggregates are more discrete in the cingulate gyrus of patient #2 (**B**), which is representative of the other cases.

We examined formalin-fixed paraffin-embedded (FFPE) brain sections according to the Brain Net standard with the paraffin-embedded tissue blot (PET blot). With this method, we detected pathologic prion protein aggregates (PrP^FFI^) in all six FFI cases. Regarding the deposition pattern of prion aggregates, a reticular/synaptic deposition pattern of pathological prion protein aggregates was present in the gray matter in all six FFI cases [8]. With the PET blot, the prion protein aggregates were visualized by a formazan reaction (purple/black color, no counter-staining) and could be scored according to distribution, deposition pattern, and density. However, the amount of PrP^FFI^ varied considerably (Figure 1). Here, the cingulate gyrus of patient #4 (Figure 1A), which displayed by far the largest amount of PrP^FFI^, and patient #2 (Figure 1B) are shown in comparison.

**Figure 2:**
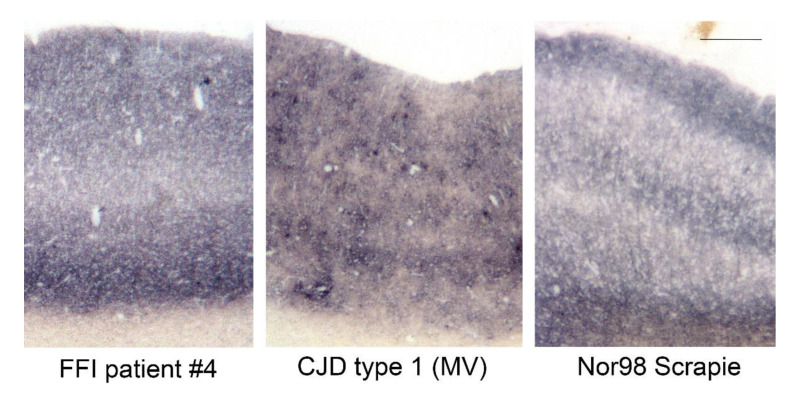
Comparison of the prion aggregate deposition pattern in human and ovine cortices stained with the PET blot method (mAb 12F10 for human tissue, mAb P4 for ovine tissue, bar = 250 µm). Prion aggregates in FFI, sporadic CJD type 1 and atypical/Nor98 scrapie are very similar and have a fine/synaptic distribution. The CJD patient (MV1) also shows fine vacuoles in the cortex (same patient as the CJD type 1 in Figure 3A).

Upon comparison with other prion diseases in humans and animals, PrP^FFI^ aggregates resemble the ones found in human sporadic CJD type 1 and atypical/Nor98 scrapie in sheep (Figure 2).

We used snap frozen tissue samples for the biochemical characterization of the prion aggregates. Table 1 provides the genotype of the six patients and the individual brain sections available for each case.

We used the sensitive membrane adsorption assay (MAA) to detect PrP^FFI^ in all available brain regions and estimated its amount for the following experiments [14]. As the detergent in the homogenization buffer plays a crucial role in the detectability of small PrP aggregates, e.g., in atypical scrapie and CJD type 1 [15], two mild detergents in different concentrations (desoxycholate and Triton X-100) were tested for FFI homogenates in comparison to phosphate-buffered saline alone (PBS pH 7.4). The detection of PrP^FFI^ was most successful with 10% homogenates in PBS with 0.5% desoxycholate; therefore, all following samples were prepared with these same procedures.

**Figure 3:**
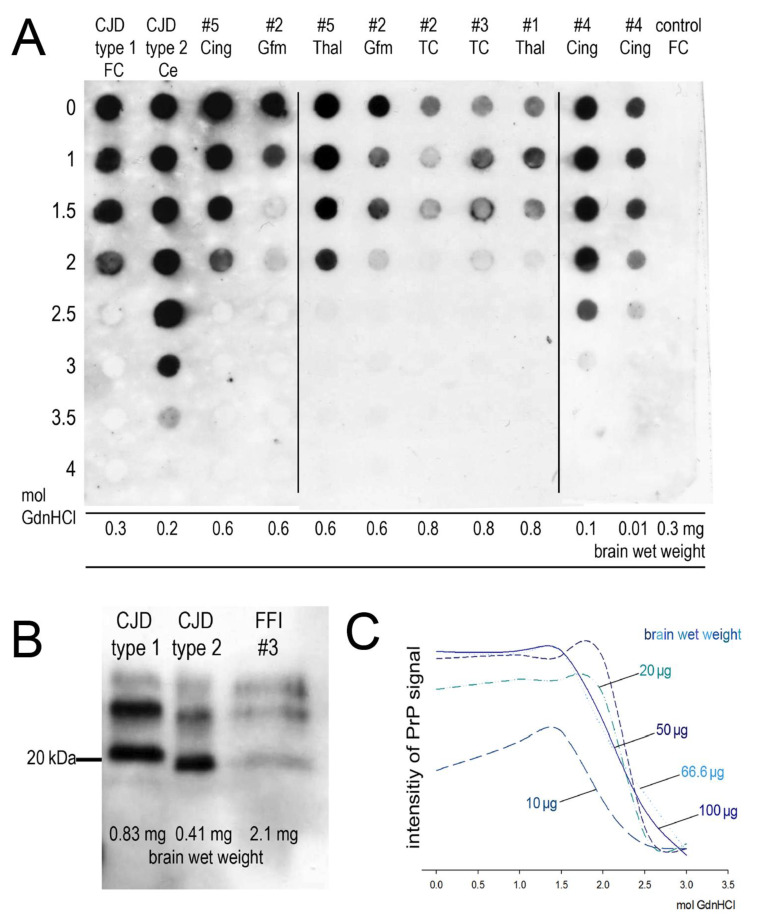
Characterization of the prion aggregates in five FFI patients: (**A**) stability of pathological prion protein against denaturation with GdnHCl. Brain regions: Ce = cerebellum, FC = Gyrus frontalis superior, Gfm = Gyrus frontalis medius, TC = Gyrus temporalis inferior, Thal = mediodorsal thalamic nucleus, Cing = Gyrus cinguli (mAb 3F4 1:3000). (**B**) Western blot profile of the pathological prion protein in sporadic CJD type 1, sporadic CJD type 2 and FFI (Gyrus frontalis superior, patient #3) (mAb 3F4 1:3000). (**C**) Densitometry of serial dilutions of PrP^FFI^ (Gyrus cinguli, brain wet weight) being tested in the stability assay (Image J; Sigma plot 11).

As shown in Table 2, the MAA detected proteinase-K-resistant PrP^FFI^ in all regions and all patients except for the cerebellum of patient #4. This finding supports other distribution studies where cerebellum and occipital cortices were the last structures to accumulate PrP^FFI^ [4]. In patient #6, only one native tissue sample could be examined. The amount of PrP^FFI^ was not always sufficient to perform a Western blot with a reasonable amount of tissue, or to determine the stability of PrP^FFI^ against denaturation with GdnHCl as described previously for sporadic CJD and ovine scrapie [12] (Table 2). Whenever a sufficient amount of PrP^FFI^ was present, Western blotting revealed the typical PrP^FFI^ pattern with strong di- and monoglycosylated fractions and an unglycosylated fraction of 19 kD, as shown in Figure 3B and documented in Table 2.

During the stability assay, homogenates were exposed to the chaotropic salt guanidine hydrochloride (GdnHCl) in ascending concentrations (1, 1.5, 2, 2.5, 3, 3.5 and 4 M). The pathological prion aggregates were thus destabilized and subsequently digested with proteinase K. We found PrP^FFI^ to be less stable than the pathological prion protein in sporadic CJD type 2 and some FFI cases, e.g., patient #5 comprises pathological prion proteins of the same stability as cases of sporadic CJD type 1 (Figure 3A). 

Patients #1, #2, and #3 likewise showed a stability of prion aggregates up to 2.0 M GdnHCl although harboring smaller amounts of PrP^FFI^ than patient #5. As the abundant PrP^FFI^ aggregates in patient #4 apparently had the highest stability, we conducted serial dilutions to rule out that stability here was a matter of quantity. Figure 3A,C shows that the brain’s wet weight needs to be reduced to a tenth in order to diminish the signal of one dot (= 0.5 mol GdnHCl). Therefore, prion aggregates in patient #4 seemed to be more stable than prion aggregates of the other four FFI cases where sufficient PrP^FFI^ was present to perform the stability assay. 

## 3. Discussion

In the current study, we characterized the prion aggregates of six FFI patients in parallel to the prion aggregate type scheme for sporadic Creutzfeldt-Jacob disease and scrapie, which helped to identify prion types across species [12]. In a similar manner, Zanusso et al. showed similarities between BSE forms (classic, H-type, and BASE) and CJD forms (variant CJD, sporadic CJD type 1 and type 2) comparing prion deposition patterns and 2D gel electrophoresis [13,16]. Prion types show type-characteristic deposition patterns and biochemical properties (i.e. stability against unfolding) that are indicative to be caused by different main conformational motifs. 

The sensitive PET blot method revealed reticular, synaptic prion aggregates in the brain regions of all FFI patients that match the prion aggregate deposition pattern of sporadic CJD type 1 and atypical/Nor98 scrapie in sheep. In all but one case, the typical FFI Western blot profile could be obtained with an unglycosylated fragment of 19 kDa as in sporadic CJD type 2. Regarding the stability of the prion aggregates, most cases seemed to fit in with CJD type 1, showing a detectable aggregate of only up to 2 M GdnHCl. Prion aggregates from one case were slightly more stable, also after 10-fold dilution, and therefore slightly different from the other four cases. It may be that FFI cases do not represent a uniform prion template for misfolded prion aggregates. Recently, two distinct prions in one Japanese FFI family were described [17]. Yet, none of the FFI cases had prion aggregates with the same stability as shown for sporadic CJD type 2. Therefore, pathological prion protein in FFI exhibits features of both human sporadic CJD types.

Among the six patients we examined, two were heterozygous at codon 129, which did not result in differences regarding the prion aggregate deposition pattern or the stability against GdnHCl (the patient with the more stable prion aggregates being homozygous). While the polymorphism at codon 129 seems to affect the clinical disease course [5], there did not seem to be a detectable difference with our experiments. Similarly, for sporadic CJD, only the type determines the stability of the prion aggregates and whether prion deposits are synaptic or complex, but not the methionine/valine polymorphism at codon 129 of the prion protein gene [12]. In sporadic CJD, the disease course and histopathological features are influenced by the codon 129 polymorphism [18], with the MM2 thalamic form mimicking FFI, which is therefore addressed as sporadic FFI [19,20].

The pathological prion in FFI could well represent a third type of human prion disease in addition to the two types in sporadic CJD. PrP^FFI^ shares the conformation around the 97th amino acid with CJD type 2, where both prion types are cleaved by proteinase K. Almost certainly it must also share a conformational motif with CJD type 1 toward the C-terminus, leading to the fine synaptic prion aggregates and lower stability against denaturation. For prion type 2, a conformational motif was described with an amyloid core, in which the side chain of the polymorphic residue at codon 129 is deeply buried [21]. Using stop mutants, Skora et al. showed how valine at codon 129 enhances the prion aggregation with type 2 properties [21]. To the best of our knowledge, for type 1 prions, such a structural motif has not yet been described.

Conformational motifs retained during transmission, as was shown by Telling et al. [22], seem to be the basis of prion diversity. Modifying factors, such as the genotype or host-specific RNAs, may lead to more than one strain in a new host of a different species upon serial transmission of one prion type. Our experiments show that a prion type needs valid criteria to indicate a specific conformational motif, and we think that proteinase K cleavage sites, the stability of the protein aggregates, and their deposition pattern could, in combination, be an appropriate approach for defining a prion type.

## 4. Materials and Methods

### 4.1. Patients

All six patients were neuropathologically diagnosed at the Prion and Dementia Research Unit of the University Medical Center Goettingen. The characteristic neuropathological changes were gliosis and spongiform changes in thalamus and subiculum, as well as a nerve cell loss and a gliosis of the nucleus olivaris inferior. In all patients, prion protein aggregates were detectable in these regions with the PET blot method. Genotyping was performed as previously described [23]. Brain sections were preserved according to the Brain Net standard in 4% paraformaldehyde, followed by embedding in paraffin (using isopropanol and xylene). In addition, snap frozen sections were preserved and kept at −80 °C. All experiments were conducted in an S3* laboratory. Three human controls (cortices from sCJD type 1 and type 2, one negative for prion) and cortex from a sheep with Nor98/atypical scrapie from a previously published study [12] were included for comparison. 

### 4.2. PET Blot 

We used the paraffin-embedded tissue blot (PET blot) on paraffin-embedded sections as described before [7,12]. In brief, sections were put on nitrocellulose membranes (0.45 µm pore size, Bio Rad, Hercules, CA, USA), dried at 80 °C for 2 days, and then deparaffinized using xylene and descending concentrations of isopropanol. A harsh proteinase K digestion (250 µg/mL in proteinase K buffer: Tris-buffered saline pH 7.6 with 0.5% Brij® 35, Roth Hydraulics, NA, USA) took place overnight at 56 °C using the pillow technique, i.e., the membranes were soaked with the proteinase K solution on non-dissolving lab wipes and stayed moist during digestion without being covered in fluid. This process was followed by rinsing the membranes in TBST, i.e. Tris-buffered saline (pH 7.6) with 0.1% Tween 20 (Carl Roth GmbH, Karlsruhe, Germany) and antigen retrieval with 4 M guanidine thiocyanate (GdnSCN, Carl Roth GmbH, Karlsruhe, Germany) took place for 15 min. Further, we conducted rinsing in TBST, blocking for 15 min with casein 0.2% (I-block, Applied Biosystems, Foster City, CA, USA) in phosphate-buffered saline pH 7.4 with 0.1% Tween 20 (PBST), and incubation with the primary anti-prion protein monoclonal antibodies, clone 12F10 (1:5000), or clone P4 (1:2000, for ovine tissue, R8008 RIDA ® Biopharm AG, Darmstadt, Germany), for 90 min. Thorough rinsing in TBST and incubation with the secondary alkaline phosphatase, coupled with goat anti-mouse antibody (0486 Dako, Glostrup, Denmark) was followed by rinsing in TBST and NTM (100 mM Tris pH 9.5, 100 mM NaCl, 50 mM MgCl_2_), and visualization with a formazan reaction using nitro-blue tetrazolium (Roche Diagnostics, GmbH, Mannheim, Germany) and bromo-chloro-indolylp hosphate (Roche Diagnostics, GmbH, Mannheim, Germany) in NTM.

### 4.3. Western Blot, Membrane Adsorption Assay, and Stability Assay

Western blot and membrane adsorption assay (MAA) were carried out as described previously [12]. We homogenized frozen tissue sections in PBS with 0.5% desoxycholate (Fluka, Buchs, Switzerland). For the MAA, DNA had to be removed using DNAse I (A3778, Applichem, Darmstadt, Germany; 5 µg enzyme per mg brain tissue, 37 °C, 30 min). The Western blot included proteinase K-digestion (50 µg/mL, 35 min) in 4× PBS pH 7.4 with 100 mM MgCl_2_ added, SDS-PAGE on 15% acrylamide gels and semi-dry blotting onto a nitrocellulose membrane (0.45 µm pore size, Bio Rad, Hercules, CA, USA). For the MAA, homogenates were subjected to proteinase K digestion (50 µg/mL, 35 min) in proteinase K buffer (see above) with 100 mM CaCl_2_ and 100 mM MgCl_2_ added. They were then applied to a nitrocellulose membrane (0.45 µm, see above) with a commercially available slot/dot blot device from BioRad (Bio-Dot^®^ and Bio-Dot SF Microfiltration Apparatus). Slot or dots were rinsed with 0.1% desoxycholate before and after the samples were sucked through. Both methods included a decontamination with 4 M GdnSCN of the membranes for 30 min, and a blocking step with 0.2% casein in PBST for 30 min before the primary antibody (anti-prion protein monoclonal antibody, AS 109–112, clone 3F4) was applied to the membranes with 0.02% casein in TBS overnight at 4 °C. The anti-mouse horseradish peroxidase-coupled secondary antibody (K4000, Agilent Dako, Santa Clara, CA, USA) was applied 1:1000 in 0.02% casein in TBS for 1.5 h. Between each step, the membranes were rinsed with TBS. To visualize the result on X-ray film (Amersham HyperfilmTM ECL, GE Healthcare, Grens, Switzerland), we used chemiluminescence reagents (SuperSignal West Femto, Thermo scientific, Pierce Biotechnology, Rockford, IL, USA) according to the manufacturer’s instructions.

To investigate the stability of the pathological prion protein in FFI in brain regions with sufficient PrP^FFI^, we followed our previously published protocol [12]. In brief, the homogenates were exposed to an ascending sequence of guanidine hydrochloride (GdnHCl, Carl Roth GmbH, Karlsruhe, Germany) dilutions for 90 min. The denatured protein homogenate was subsequently digested with proteinase K, and the MAA was carried out as usual (see above). 

We determined the density of the dots on X-ray film using Image J and plotted the result with Sigma plot 11. 

## Figures and Tables

**Table 1:** Frozen tissue available for biochemical characterization, i.e., Western blot analysis and stability assay against GdnHCl using a membrane adsorption assay (MAA). The codon 129 polymorphism of the prion protein gene is provided for each case (homozygosity for methionine (MM) or heterozygosity (methionine/valine MV)).

Case Number	Codon 129 Polymorphism	Brain Regions Available for Homogenates
#1	MV	Gyrus cinguli, Gyrus temporalis inferior, Gyrus frontalis medius, Gyrus frontalis superior, mediodorsal thalamic nuclei
#2	MM	Gyrus cinguli, Gyrus temporalis inferior, Gyrus frontalis medius, Gyrus frontalis superior
#3	MM	Gyrus temporalis inferior, Gyrus frontalis superior
#4	MM	Gyrus cinguli, Gyrus temporalis inferior, Gyrus frontalis medius, Gyrus frontalis superior, mediodorsal thalamic nuclei, Nucleus caudatus, Cerebellum
#5	MV	Gyrus cinguli, Gyrus temporalis inferior, Gyrus frontalis medius, Gyrus frontalis superior, mediodorsal thalamic nuclei, Nucleus caudatus, Cerebellum
#6	MM	Gyrus frontalis superior

**Table 2:** Summary of results for each available native tissue sample. First, we applied a membrane adsorption assay (MAA) to estimate the amount of PrP^FFI^. If a sufficient amount of PrP^FFI^ was present, homogenates were subjected to Western blot analysis and the stability assay.

Case Number	Brain Area	MAA	Stability MAA	Western Blot
#1	Gyrus cinguli	positive	not possible	n.d.
	Gyrus temporalis inferior	positive	not possible	n.d.
	Gyrus frontalis medius	positive	not possible	n.d.
	Gyrus frontalis superior	positive	not possible	not possible
	mediodorsal thalamic nuclei	positive	possible	typical FFI signature
#2	Gyrus cinguli	positive	not possible	n.d.
	Gyrus temporalis inferior	positive	possible	n.d.
	Gyrus frontalis medius	positive	possible	typical FFI signature
	Gyrus frontalis superior	positive	possible	typical FFI signature
#3	Gyrus temporalis inferior	positive	possible	typical FFI signature
	Gyrus frontalis superior	positive	possible	typical FFI signature
#4	Gyrus cinguli	positive	possible	typical FFI signature
	Gyrus temporalis inferior	positive	possible	n.d.
	Gyrus frontalis medius	positive	possible	n.d.
	Gyrus frontalis superior	positive	possible	typical FFI signature
	mediodorsal thalamic nuclei	positive	possible	n.d.
	Nucleus caudatus	positive	possible	n.d.
	Cerebellum	negative	not possible	not possible
#5	Gyrus cinguli	positive	possible	typical FFI signature
	Gyrus temporalis inferior	positive	possible	typical FFI signature
	Gyrus frontalis medius	positive	possible, but not sufficient for final determination of stability	n.d.
	mediodorsal thalamic nuclei	positive	possible	n.d.
	Nucleus caudatus	positive	not possible	n.d.
	Cerebellum	positive	not possible	n.d.
#6	Gyrus frontalis superior	positive	not possible	not possible

Note: ‘n.d.’—not done.
